# Effects of a Psychological First Aid (PFA) Based on the SIX Cs Model on Acute Stress Responses in a Simulated Emergency

**DOI:** 10.1192/j.eurpsy.2026.11336

**Published:** 2026-07-31

**Authors:** M. Farchi

**Affiliations:** Social Work, Tel-Hai University on the rise, Upper Galilee, Israel

## Abstract

**Introduction:**

הנה גרסה מקוצרת ל־**רקע תיאורטי** בהיקף של כ־300 תווים:

Acute stress reactions (ASR) impair functioning and elevate PTSD risk. Traditional early interventions, like psychological debriefing, proved ineffective. The SIX Cs model offers a cognitive, action-oriented PFA approach—enhancing control, resilience, and recovery while reducing dependence on professionals.

**Objectives:**

This study aimed to test the effectiveness of the SIX Cs model in mitigating acute stress. Objectives were to assess its impact on anxiety, resilience, and HRV, and to examine whether it promotes faster recovery compared to emotion-focused support.

**Methods:**

This study demonstrates that the SIX Cs model of Psychological First Aid effectively reduces acute stress reactions and accelerates recovery compared to emotion-focused support. Participants receiving the intervention showed preserved resilience, stabilized heart rate variability, and lower anxiety. These findings emphasize the value of cognitive activation and structured engagement immediately after exposure to extreme stress. The model’s brevity and simplicity make it accessible for nonprofessionals and first responders, offering a scalable and evidence-based tool to strengthen coping, reduce PTSD risk, and enhance community resilience in emergencies.

**Results:**

This study demonstrates that the SIX Cs model of Psychological First Aid effectively reduces acute stress reactions and accelerates recovery compared to emotion-focused support. Participants receiving the intervention showed preserved resilience, stabilized heart rate variability, and lower anxiety. These findings emphasize the value of cognitive activation and structured engagement immediately after exposure to extreme stress. The model’s brevity and simplicity make it accessible for nonprofessionals and first responders, offering a scalable and evidence-based tool to strengthen coping, reduce PTSD risk, and enhance community resilience in emergencies.

**Image 2:**
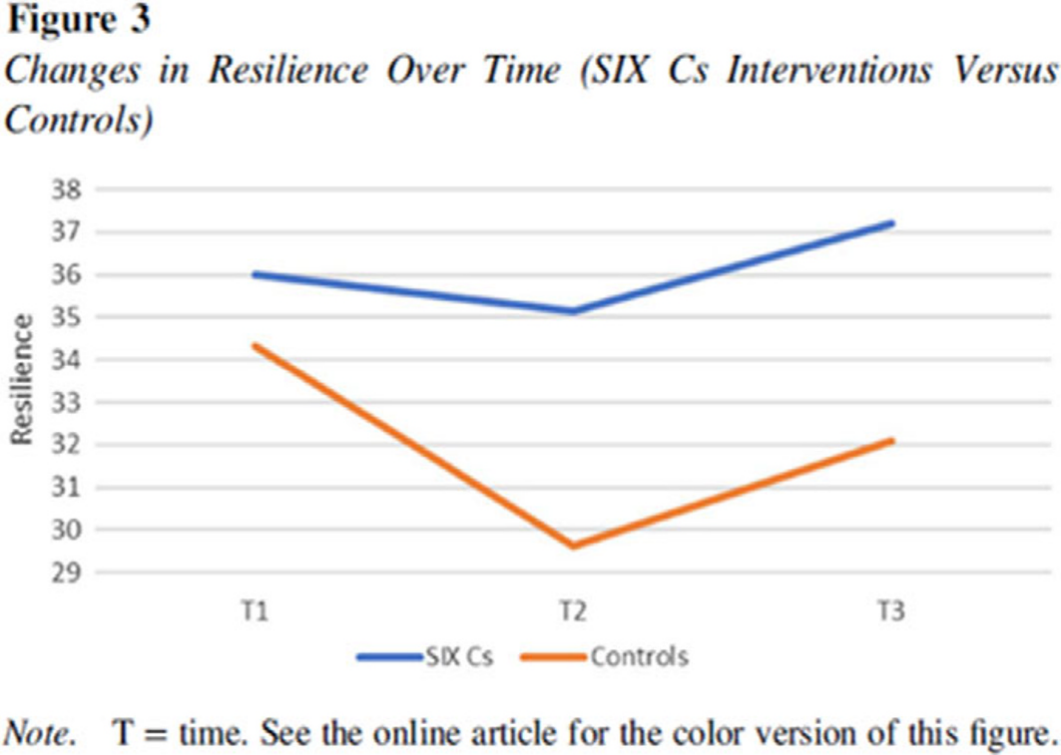
Image 2: Long description.

**Image 3:**
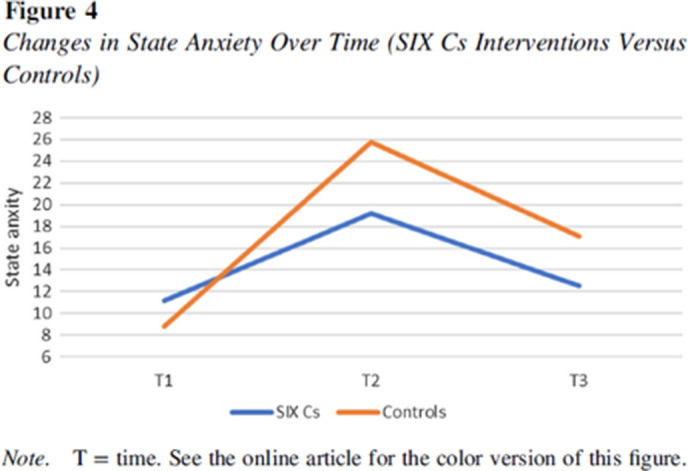
Image 3: Long description.

**Conclusions:**

The study demonstrates that the SIX Cs model of Psychological First Aid effectively reduces acute stress reactions and accelerates recovery compared to emotion-focused support. Participants receiving the intervention showed preserved resilience, stabilized heart rate variability, and lower anxiety. These findings emphasize the value of cognitive activation and structured engagement immediately after exposure to extreme stress. The model’s brevity and simplicity make it accessible for nonprofessionals and first responders, offering a scalable and evidence-based tool to strengthen coping, reduce PTSD risk, and enhance community resilience in emergencies.

**Disclosure of Interest:**

None Declared

## Long descriptions

### Image 2: Long description

The Y-axis is labeled Resilience with values ranging from 29 to 38 in increments of 1. The X-axis represents time with three points labeled T 1, T 2, and T 3.

* The SIX Cs group (blue line) starts at a resilience score of 36 at T 1, shows a slight decrease to approximately 35.1 at T 2, and then increases to a peak of approximately 37.2 at T 3.

* The Controls group (orange line) starts at a resilience score of approximately 34.3 at T 1, drops sharply to a low of approximately 29.6 at T 2, and then recovers to approximately 32.1 at T 3.

A legend at the bottom indicates the blue line represents SIX Cs and the orange line represents Controls. A note below the graph clarifies that T equals time.

Navigate back to Image 2:.

### Image 3: Long description

The Y-axis is labeled State anxiety with numerical increments from 6 to 28. The X-axis represents time with three points labeled T 1, T 2, and T 3. A legend at the bottom identifies two lines: a blue line for S I X Cs and an orange line for Controls.

* The S I X Cs group (blue line) starts at approximately 11 at T 1, rises to a peak of roughly 19 at T 2, and decreases to approximately 12.5 at T 3.

* The Controls group (orange line) starts lower at approximately 9 at T 1, rises sharply to a higher peak of approximately 26 at T 2, and decreases to approximately 17 at T 3.

Both groups show a similar trend of increasing anxiety from T 1 to T 2 followed by a decrease at T 3, but the Controls group maintains higher anxiety levels at T 2 and T 3 compared to the S I X Cs group.

Navigate back to Image 3:.

